# CD4+T cell metabolic reprogramming as therapeutic targets in neurodegenerative diseases

**DOI:** 10.1186/s13041-026-01282-6

**Published:** 2026-02-25

**Authors:** Prince Amoah Barnie, Karen Pomeyie, Benjamin Amoani, Foster Kyei, Abdala Ussif Mumuni, Justice Afrifa, Samuel Essien-Baidoo, Daniel Boison

**Affiliations:** 1 Department of Forensic Sciences, School of Biological Sciences, College of Agriculture and Natural Sciences, University of Cape Coast, Cape Coast, Ghana; 2 International Genome Centre, Jiangsu University, Zhenjiang, 212013 Jiangsu Province People’s Republic of China; 3 Department of Immunology, Jiangsu University, Zhenjiang, 212013 Jiangsu Province People’s Republic of China; 4 Virology Department, Noguchi Memorial Institute for Medical Research, University of Ghana, Accra, Ghana; 5 Department of Biomedical Sciences, School of Allied Health Sciences, University of Cape Coast, Cape Coast, Ghana; 6 Department of Molecular Biology & Biotechnology, School of Biological Sciences, College of Agriculture and Natural Sciences, University of Cape Coast, Cape Coast, Ghana; 7 Department of Medical Laboratory Science, School of Allied Health Sciences, University of Cape Coast, Cape Coast, Ghana; 8 Scientific Research Centre, Second Affiliated Hospital of Harbin Medical University, Harbin, China; 9 Department of Biochemistry, School of Biological Sciences, College of Agriculture and Natural Sciences, University of Cape Coast, Cape Coast, Ghana

**Keywords:** Neurodegenerative diseases, Metabolic reprogramming, CD4+T cells, Treg, Autoimmunity

## Abstract

Neurodegenerative diseases are a group of disorders characterized by the progressive loss of structure and function of neurons in the brain and/or peripheral nervous system. The main pathological feature of neurodegenerative disease in the central nervous system (CNS) is the selective neuronal loss in the brain and spinal cord, leading to cognitive and/or motor dysfunction. The immune system plays a variety of roles in the pathophysiology of neurodegenerative diseases. CD4+T cells are being recognized as important immunometabolic modulators in the pathophysiology of neurodegenerative disorders (ND), including multiple sclerosis (MS), Parkinson’s disease (PD), and Alzheimer’s disease (AD). Their varied metabolic patterns provide a special therapeutic window for regulating neuroinflammation, spanning from lipid-dependent regulatory T cells (Tregs) to glycolysis-driven pro-inflammatory subsets (Th1, Th17). Abnormal immune metabolism raises the risk of oxidative stress, mitochondrial malfunction, and neuronal death in neurodegenerative environments. According to recent research, altering CD4 T cell metabolism to favour oxidative phosphorylation (OXPHOS) and fatty acid oxidation (FAO) may help Treg function return and inhibit harmful effector responses. Current research on CD4 T cell immunometabolic pathways, their interactions with CNS-resident cells, and the developing possibility of metabolic intervention to slow neurodegeneration is explained in this review. By examining important signaling pathways including AMPK, mTORC1, and ROS dynamics, we demonstrate how CD4+T cell metabolism may reshape ND treatment approaches.

## Introduction

Neurodegenerative disorders are characterized by glial activation and an enhanced proinflammatory microenvironment, leading to the loss of neurons [28]. The exact initiating mechanisms remain unclear, but aging is considered the principal risk factor. In diseases like Alzheimer’s disease (AD), multiple sclerosis (MS), Parkinson’s disease (PD), and AIDS dementia, increased neuronal apoptosis occurs due to an unregulated inflammatory response [18]. Another key feature of neurodegenerative diseases is synaptopathy, where there is a reduction in synapse numbers and function, often preceding neuronal loss and cell death [6]. Neuroinflammation in neurodegenerative diseases begins with the increased expression and release of proinflammatory molecules such as TNFα, IL1β, and nitric oxide from glial cells [73]. Inflammatory factors like TNF-alpha, IL-1 beta, and IL-6 interact with reactive oxygen species (ROS) in a detrimental feed-forward loop that disrupts normal metabolic processes, ultimately driving neurodegeneration (Omeye Francis). Despite the crucial role neuroinflammation plays in the progression of these diseases, anti-inflammatory drugs have not shown significant therapeutic effects in PD, AD, and MS patients [24]. Other therapeutic agents currently used can only alleviate symptoms temporarily and become ineffective with prolonged use, without curing the disease [21]. As a result, identifying therapeutic strategies that can prevent, halt, or cure neurodegenerative diseases has become a major focus in recent years. Targeting the adaptive immune system has emerged as a promising approach due to its involvement in the pathogenesis of these diseases, and its potential to modulate neuroinflammation has been a critical process in neurodegeneration. Researchers now speculate that targeting immune-metabolic interactions could represent the next frontier in therapeutic strategies for neurodegenerative diseases [52]. Compelling findings in the field of immunometabolism highlight how the metabolic status of immune cells can influence various immune responses, potentially offering new avenues for treatment [1]. In the pathogenesis of neurodegenerative disorders (ND), various immune and non-immune cells cross the blood–brain barrier (BBB) and are recruited into the central nervous system (CNS) [56]. These infiltrating cells, such as T-lymphocytes, neutrophils, and resident microglia, contribute to the neuroinflammation observed in these diseases [76]. Microglia, as the first line of defense against pathogens, play a crucial role in initiating responses to injury in the CNS. Their activation, along with the involvement of astrocytes and infiltrating peripheral immune cells, creates a neuroinflammatory cycle that promotes neurodegeneration [49]. In particular, infiltrating T cells in the CNS are implicated in the elevated frequencies of Th17 cells and other T lymphocytes observed in postmortem PD brain tissues [84]. These T lymphocytes mediate neuronal cell death in PD through IL-17 signaling via the IL-17 receptor. Th17 cells induce microglial activation and production of pro-inflammatory cytokines like IL-1β, TNF-α, and IL-6. These infiltrating lymphocytes and neutrophils exacerbate oxidative stress and mitochondrial dysfunction in PD, although the exact contribution of reactive oxygen species (ROS) generated by these cells to neurodegeneration remains unclear [26]. It is reported that ROS production in T cells increases as a result of metabolic reprogramming to OXPHOS, during T cell activation, thus, contributing to mitochondrial dysfunction and oxidative damage in ND [68]. Recent studies also suggest that metabolic reprogramming in neurodegenerative diseases leads to the generation of ROS and the disruption of ATP production [58]. This metabolic change is often associated with inefficient glucose metabolism in neurons, which, along with oxidative stress, is a key driver of neuronal cell death [51]. Glucose and cholesterol metabolism have been shown to significantly influence the pathogenesis of various ND [9]. In AD, for instance, disruptions in glucose uptake and metabolism are evident, and elevated amyloid-beta (Aβ) proteins are observed. Aβ proteins contribute to neurotoxicity through intracellular signaling pathways that increase intracellular calcium levels and activate kinases and caspases [9]. Additionally, Aβ proteins affect cholesterol metabolism in neurons, leading to reduced cholesterol levels, hyperphosphorylation of tau, and neurodegeneration [51]. It can be inferred that the decreased levels of cholesterol may be associated with de-myelination of the myelin sheath of neurons [8]. This implies that the protective barrier of fragile nerve barriers has been removed, nutrient exchange may be impeded and consequentially decreased impulse transmission, which is very characteristic of neurodegeneration [70]. In response to stimuli like lipopolysaccharides (LPS), microglia undergo a metabolic shift from oxidative metabolism to glycolysis, similar to other immune cells [48]. This process of metabolic reprogramming is receiving increasing attention in the context of cancers and autoimmunity [93]. Specifically, reprogramming the metabolism of activated CD4+T cells from glycolysis to FAO may offer therapeutic potential in mitigating the severity of neurodegenerative diseases [53]. This review highlights the communication between CD4+T cells, resident immune cells, and non-immune cells in the development of ND. It also discusses how metabolic reprogramming of CD4+T cells could serve as a promising therapeutic strategy in the management of these diseases. Ultimately, the article highlights the potential of targeting metabolic remodeling in immune cells as a novel approach to treating neurodegenerative disorders, providing valuable insights into the future of neurodegenerative therapy.

### T cell subtypes and their properties

T cells are a diverse group of lymphocytes central to the adaptive immune system, each with specialized functions and properties defined by specific cell surface markers and secreted cytokines. The major subtypes are helper, cytotoxic, regulatory, and memory T cells [10]. Functionally, T helper (Th) cells secrete cytokines to activate B cells, cytotoxic T cells and macrophages [34]. They differentiate into further subsets such as Th1, Th2, Th17, Gamma Delta and natural killer based on the cytokine environment [46]. Cytotoxic T Lymphocytes (CTLs) recognize antigens on MHC Class I molecules (found on most nucleated cells); release perforin and granzymes to induce apoptosis (programmed cell death) in target cells [12]. Regulatory T (Treg) Cells Secrete inhibitory cytokines like IL-10 and TGF-β; prevent autoimmune diseases and overactive immune reactions [62]. Memory T Cells are long-lived cells that respond rapidly and vigorously to a second encounter with the same antigen, quickly becoming effector cells. Subtypes of memory T cells include central memory (Tcm) and effector memory (Tem) cells [59].

### CD4+T cell metabolism in neurodegeneration

**Fig. 1:**
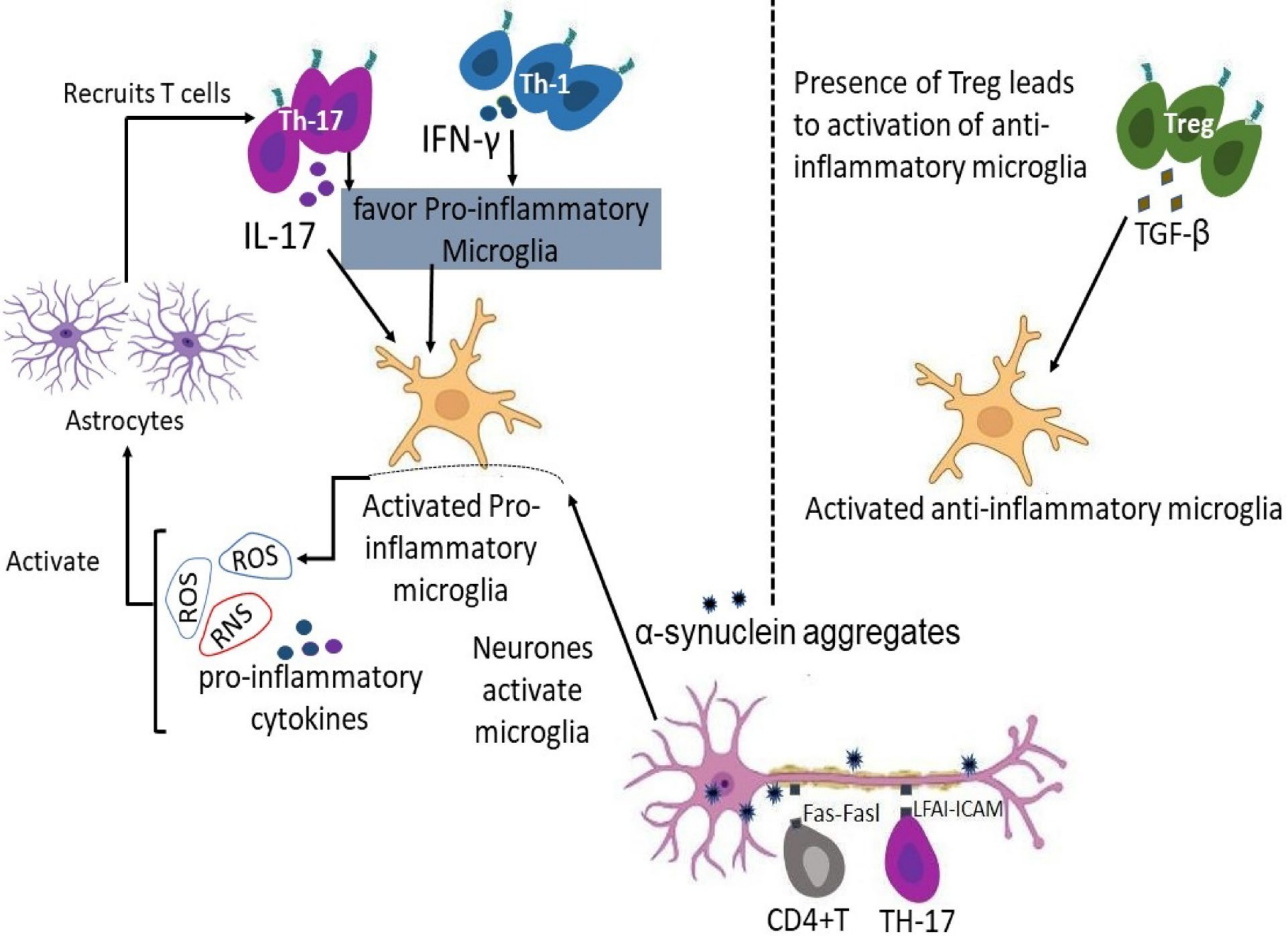
CD4+T Cell activation, recruitment and functions in the pathogenesis of neurodegenerative diseases. Activation of microglia by neuron increases ROS, RNS and proinflammatory cytokines, which activates astrocytes to influence the recruitment of T cells into the CNS. The presence of T cells could to a large extent determine the fate of activated microglia in a diseased brain. The Bidirectional crosstalk between T cells and neuron influences T cells differentiation into effector T cells. Treg stimulates a shift towards the anti-inflammatory microglia which is predominantly anti-inflammatory in nature. TH-17 and TH-1 stimulates a shift towards the pro-inflammatory microglia which is pro-inflammatory in nature. These CD4+T cells release inflammatory molecules, contributing to neuronal damage. Theses CD4+T cells can directly kill neurons through pathways like the Fas/FasL pathway. α-synuclein aggregates into toxic clumps (lewy bodies) and contributes to neuronal death

CD4⁺ Depending on how their metabolism is set up, T cells can either drive harm or encourage tissue repair in neurodegenerative illnesses [50]. Pro-inflammatory subsets of T cells that depend on glucose and glutamine metabolism are frequently seen in Parkinson’s disease, multiple sclerosis, and Alzheimer’s disease [38]. This propensity for glycolysis promotes the quick development and release of harmful cytokines [82]. Meanwhile, mitochondrial stress and altered lipid utilization cause regulatory T cells (Tregs), which normally reduce inflammation and support central nervous system health, to lose their functional stability. The course of the illness is exacerbated by this imbalance between effector and regulatory cell states [13] (Fig. 1).

## Immunometabolism of T cells in migration and CNS infiltration

T lymphocytes’ ability to successfully migrate across the blood–brain barrier (BBB) and into the central nervous system (CNS) is controlled by the metabolic programming of the cells as well as chemokine signaling and adhesion interactions [80]. For T cells to adapt to the mechanical challenges of transmigration, changing oxygen levels, and restricted food supply along the migratory route, their metabolism must be dynamically realigned [33]. To support the cytoskeletal remodeling and membrane dynamics necessary for movement, effector T cells, in particular, increase glycolytic flux [91]. The process of mitochondrial OXPHOS, which produces ATP continuously, facilitates both endothelium traversal and chemotaxis [47]. During migration, distinct subsets of T cells adopt specific metabolic strategies [43]. Fatty acid oxidation is used by memory and regulatory T cells to preserve mitochondrial robustness and energy efficiency throughout extended transit [72]. In situations where nutrients are scarce, this metabolic pathway is particularly important for Treg migration and function [40]. The immunometabolic environment that invading T lymphocytes encounter upon entering the central nervous system is influenced by hypoxia, reactive oxygen species (ROS), and limited availability of glucose and glutamine [27]. T cells need to quickly modify their metabolic pathways to survive and operate in this setting [11]. While effector populations of CD4+and CD8+rely on ongoing glycolysis and biosynthetic flux to generate inflammatory responses, regulatory T cells depend on OXPHOS and FAO to maintain their suppressive nature [75]. Thus, T cells’ immunometabolic profile is closely related to how they coordinate migration and CNS residence [33]. Gaining knowledge of these pathways can help develop treatment plans that alter T cell activation and trafficking in neuroinflammatory and neurodegenerative illnesses [55].

### Metabolic dependency of CD4+ T cell differentiation in neuroinflammation

The way a naïve cell’s internal metabolism is rewired after activation determines whether it develops into a pro-inflammatory or regulatory CD4+T cell [25]. Important intracellular regulators as well as extrinsic cues like cytokines are responsible for this metabolic change [15]. Cytokine production and clonal proliferation are made possible by glycolytic reprogramming toward effector Th1 and Th17 fates, which is driven by the mTORC1 and HIF-1α pathways [63]. On the other hand, AMPK activation encourages fatty acid OXPHOS, a slower and more durable metabolic state that supports Foxp3 stability and Treg differentiation [40]. This metabolic bifurcation identifies a cellular control point for potential treatments and supports the way T cells select their functional pathways during neuroinflammation [83]. Foxp3 and AMPK function as critical synergistic regulators that maintain immune tolerance and provide neuroprotection by controlling the metabolic and suppressive identity of regulatory T cells [92]. Activating the AMPK-FOXO3 pathway induces antioxidant systems that protect neurons from reactive oxygen species (ROS). AMPK activation in microglia again inhibits NF-κB signaling, shifting them from a pro-inflammatory to an anti-inflammatory state [67].

## Metabolic reprogramming and crosstalk of CD4 T cell pathways in neurodegeneration

**Fig. 2:**
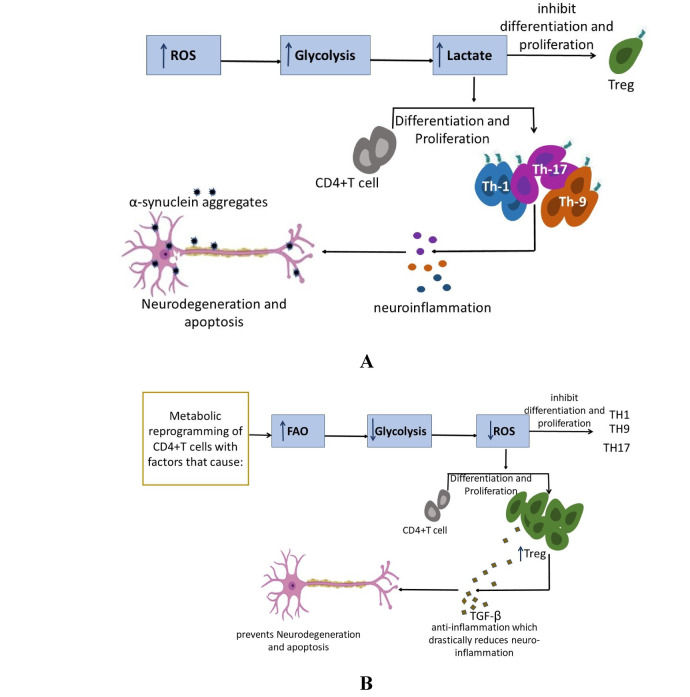
Metabolic reprogramming of CD4+T cell in neurodegenerative diseases. Metabolic processes modulate the differentiation, proliferation and function of CD4+T cells. The metabolic conditions enable CD4+T cells to function either as neurotoxic or neuroprotective. **A** High levels of ROS, increased Glycolysis and high concentration of lactate promote the proliferation of Th1, Th9 and Th17 cells, which promote neuroinflammation and leads to neurodegeneration and apoptosis of neurons. **B** Metabolic reprogramming of CD4+T cells with a therapy or intervention, promotes FAO metabolism, reduce glycolysis and decrease ROS subsequently proliferation of T regulatory cells which ameliorate neuroinflammation and ultimately support neuronal survival

In the pathophysiology of neurodegenerative disorders, the interaction between immunometabolism and neuroinflammation has become more complicated [14]. CD4 T cells have been identified as important regulators of both neuroprotection and neurodegeneration [55]. In Parkinson’s disease, multiple sclerosis, and Alzheimer’s dementia, their metabolic reprogramming and context-dependent interactions with glial cells and peripheral immunological components have a significant impact on the course of the illness [90]. In Parkinson’s disease, dopaminergic neuronal loss in the substantia nigra has been associated with pro-inflammatory Th1 and Th17 cell peripheral activation and CNS infiltration [84]. Through a glycolytic shift driven by mTORC1 signaling, these cells produce more IFN-γ and IL-17, which intensifies neuronal stress and microglial activation [45]. Interestingly, metabolic stress in the midbrain, which is typified by elevated oxidative stress and mitochondrial dysfunction, both compromises the stability of regulatory T cells (Treg) and increases effector T cell activation [13]. Although FAO and OXPHOS are essential for the suppressive function of Tregs in Parkinson’s disease, their numbers and metabolic fitness are reduced, which compromises neuroprotective processes [35]. One of the hallmarks of the initiation and recurrence of multiple sclerosis, a classic autoimmune demyelinating illness, is CD4 T cell infiltration across the blood–brain barrier [77]. Th17 cells rely largely on aerobic glycolysis and glutaminolysis and are especially harmful. Restricting glycolysis or increasing FAO in T cells has been shown to reduce CNS inflammation and illness severity in experimental autoimmune encephalomyelitis (EAE) models [71]. In contrast, MS patients’ CNS-infiltrating Tregs show decreased Foxp3 expression and compromised mitochondrial metabolism, which are associated with a restricted ability to control inflammation [38]. In this situation, AMPK activation may enhance Treg resistance to CNS-induced metabolic limitations and partially restore mitochondrial biogenesis [22]. The neuroinflammatory milieu produced by amyloid-beta plaque buildup and persistent microglial activation in AD makes it easier for CD4 T cells to be recruited and reprogrammed [42]. T cells trying to operate inside the cortical and hippocampal areas are subjected to high energy demands due to elevated ROS levels and reduced nutritional availability, specifically glucose and glutamine [75]. The production of cytokines and the promotion of antigen presentation by glycolysis-driven Th1 and Th17 cells may intensify local inflammation [78]. On the other hand, Tregs with intact mitochondrial respiration are linked to better cognitive outcomes and a lower plaque load in mouse models, highlighting the need to enhance Treg metabolism with focused therapies [97]. Enhancing oxidative pathways or increasing the production of mitochondrial enzymes through transcriptional programs regulated by Foxp3 may offer therapeutic prospects in the immunomodulation of AD [31]. Together, these diseases show that CD4 T cell metabolic flexibility determines their destiny and function in neurodegenerative environments rather than being a simple consequence of immune activation, as reported [19]. Targeting metabolic checkpoints, including AMPK, ROS signaling axis, and mTOR, may help alter CD4 T cell responses in a way that promotes neuroprotection [13]. In PD, MS, and AD, future approaches that restore Treg activity while limiting effector T cell glycolysis may be able to stop or reverse the course of neurodegeneration [55] (Fig. 2).

### The impact of neuroinflammatory environment on T cell metabolism and differentiation

The physiologically hostile environment caused by neuroinflammation is characterized by low oxygen, reactive oxygen species (ROS), and nutritional deficiencies [57]. As CD4 T cells approach the central nervous system, these factors put tremendous pressure on them, which affects how they develop [41]. Signals including ROS, IL-1β, and IL-6 speed up the breakdown of glucose, which promotes the growth of Th17 cells that worsen tissue damage [2]. Treg survival is simultaneously decreased and their regulatory activities are suppressed by mitochondrial malfunction and toxic metabolites including lactate and succinate [97]. Glycolysis-dependent effector T cells have a metabolic advantage which potentially promotes their survival and proliferation, over oxidative Tregs due to competition for resources between immunological and glial cells [54]. A skewed immunological milieu that promotes chronic inflammation and quickens neurodegeneration is the end effect [95].

## Parkinson’s disease: CD4+T cell metabolic dysregulation

In Parkinson’s disease, CD4+T cells exhibit altered metabolism and immune responses, potentially contributing to neurodegeneration. These changes can manifest as shifts in T cell subsets, cytokine production, and interactions with other brain cells like astrocytes and endothelial cells. Mitochondrial failure, oxidative stress, and neuroinflammation create a physiologically unfriendly environment for CD4+T lymphocytes entering the central nervous system [81]. Studies have shown changes in the proportion of CD4+and CD8+T cells in Parkinson’s patients, with some studies reporting decreased CD4+T cells and increased CD8+T cells, leading to a lower CD4/CD8 ratio [36]. These circumstances tilt the metabolic programming of T cells in favour of pro-inflammatory characteristics [20]. Specifically, increased ROS and compromised mitochondrial respiration in peripheral immune cells, such as CD4 T cells, have been linked to a move toward glycolysis, a characteristic of Th1 and Th17 effector subsets involved in the pathophysiology of Parkinson’s disease [13]. In Parkinson’s disease models, CD4 T cells have elevated glucose transporter (GLUT1) activity and upregulated glycolytic gene expression, which promotes IFN-γ and IL-17 release that worsens dopaminergic neuron loss [32]. On the other hand, Parkinson’s disease may cause metabolic suppression of regulatory T cells (Tregs), which depend on FAO and OXPHOS [4]. This potentially results in an upsurge of pro-inflammatory factors and thus decreased neuroprotection.

### Multiple sclerosis: metabolic rewiring of CD4⁺ T cells

Significant CD4 T cell metabolic abnormalities are a hallmark of multiple sclerosis [66]. In MS, CD4+T cells are considered a major driver of the autoimmune response and their involvement is more complex, with both pathogenic and potentially beneficial roles. Metabolic changes in CD4+T cells, particularly in their mitochondria, are linked to the development and progression of MS. Hypoxia-inducible factor 1-alpha (HIF-1α) and mTORC1 activation cause Th17 activation, which are essential to MS pathogenesis, to display increased glycolysis and glutaminolysis [5]. Axonal damage and CNS demyelination are facilitated by these metabolic pathways, which also promote Th17 cell development, survival, and IL-17 release [79]. On the other hand, Tregs in MS patients have compromised mitochondrial respiration and FAO, which reduces their immunosuppressive capabilities [35]. This implies that, Tregs fail to effectively suppress the destructive effects of self-reactive cells. According to studies, T cells from MS patients exhibit impaired mitochondrial electron transport chain complex function and decreased basal and maximum oxygen consumption rates [16]. In experimental autoimmune encephalomyelitis models, therapeutic approaches that block glycolysis (e.g., 2-deoxy-D-glucose) or stimulate FAO and mitochondrial biogenesis (e.g., PGC-1α activators) can rebalance CD4+T cell subsets and lessen the severity of the disease [39].

## Alzheimer’s disease: CD4+T cell metabolic plasticity in neuroinflammation

In Alzheimer’s disease, CD4 T cell metabolism is altered, contributing to neuroinflammation and disease progression. These changes include shifts in T cell subsets, such as increased Th17 and Th1 responses, which can exacerbate the inflammatory environment in the brain. Furthermore, disruptions in glucose metabolism within the brain, potentially linked to the presence of amyloid plaques, can impact T cell function and their ability to regulate the immune response. Aβ buildup, mitochondrial ROS, and metabolic stress are indicators of the neuroinflammatory milieu in AD, which alters CD4+T cell metabolism in ways that affect the course of the illness [81]. Evidence indicates that Tregs, which are dependent on FAO and OXPHOS, provide neuroprotective benefits through IL-10 production and regulation of microglial activation, even if effector CD4+ T cells may adopt glycolysis-driven profiles that promote inflammation [35]. According to studies conducted in AD mice models Tregs’ suppressive effectiveness may be limited in late-stage illness due to lower expression of fat oxidation enzymes and poor mitochondrial respiration. By altering cytokine signaling and causing oxidative stress, Aβ itself may indirectly rewire T cell metabolism [88]. Oxidation could lead to the formation of oxidized forms of Aβ, destabilization of the Aβ aggregation processes [74] which potentially slows down the formation of the regular Aβ fibrils and consequentially decreases it pro-inflammation. Restoring Treg protective activities and providing a route toward immunometabolic treatment by restoring proper glucose metabolism in the brain in AD may be possible by improving their metabolic fitness with AMPK agonists, Foxp3 stability, or mitochondrial support [54].

### Regulation of CD4⁺ T cell immunometabolism as a therapeutic strategy in neurodegenerative diseases

Generally, metabolic targeting offers a more sophisticated approach to sculpting immune responses, potentially providing better efficacy in specific diseases with an improved safety profile compared to the broad effects of traditional cytokine blockade [85]. Targeting immunometabolic pathways in CD4⁺ T cells has emerged as a promising approach for modulating neuroinflammation in neurodegenerative diseases (NDs) [29]. Modulating T cell metabolism allows for the targeting of specific immune cell subsets (e.g., pathogenic Th17 cells) while sparing others (e.g., regulatory T cells or memory T cells crucial for immunity) [86]. This contrasts with broad anti-cytokine therapies, which suppress the entire immune response mediated by those cytokines. CD4⁺ T cell subsets including Th1, Th17 and Tregs, undergo distinct metabolic reprogramming during activation and differentiation, and these pathways are dysregulated in the inflammatory environments of PD, MS, and AD [20]. In Parkinson’s disease, CD4⁺ T cells exhibit increased glycolytic activity and oxidative stress, favoring Th1 and Th17 polarization. Elevated ROS in PD patients correlates with IFN-γ and IL-17 production, exacerbating neurodegeneration [13]. Enhancing FAO in CD4⁺ T cells can promote Treg generation and suppress pro-inflammatory responses, representing a potential strategy to rebalance the immune landscape in PD [54]. In multiple sclerosis, hyperactivated Th17 cells display a glycolysis-dependent metabolic profile driven by mTORC1 and HIF-1α signaling [94]. This enhances IL-17 secretion and facilitates CNS infiltration. Meanwhile, Tregs in MS patients have impaired mitochondrial respiration and FAO, weakening their suppressive capacity [94]. Therapeutic interventions that restore mitochondrial function or inhibit glycolysis (e.g., with 2-deoxyglucose or metformin) have shown efficacy in animal models of MS [44].

In Alzheimer’s disease, Tregs contribute to neuroprotection via IL-10-mediated suppression of microglial activation [69]. However, mitochondrial dysfunction and energy stress in the AD brain may impair Treg metabolism and survival [7]. Promoting oxidative phosphorylation and stabilizing Foxp3 expression in Tregs, via agents like AMPK activators, could strengthen their anti-inflammatory effects in AD models [97]. Metabolic manipulation of CD4+T cells can also regulate their interaction with other CNS-resident immune cells. For example, depriving CD4+T cells of glutamine favors Foxp3⁺ Treg differentiation while suppressing Th1 responses, offering a route to reshape inflammatory dynamics in the CNS [83]. Similarly, targeting fatty acid metabolism can limit Th17 differentiation and IL-17 production, which are particularly pathogenic in MS and PD [26]. Shifting activated CD4+T cells from glycolysis toward FAO not only enhances Treg fitness but also reduces effector T cell-mediated neurotoxicity [83]. Such strategies, by restoring metabolic balance and functional diversity within the CD4⁺ T cell pool, have the potential to slow neurodegenerative progression and improve clinical outcomes [29].

Researchers have made significant progresses in modulating T cell metabolism in the treatment of inflammatory conditions. Using AMPK activators or mTORC1 inhibitors to encourage a metabolic shift in CD4 T cells from glycolysis to FAO has demonstrated potential in preclinical models for boosting Treg responses and reducing PD-associated neuroinflammation [23]. Research by Park et al. [64] investigated the neuroprotective effects of osmotin using in vitro and in vivo models of PD. The authors documented that osmotin alleviated the accumulation of α-synuclein by promoting the AMPK/mammalian target of rapamycin (mTOR) autophagy signaling pathway and further concluded that suggest that osmotin has potential neuroprotective effects in PD neuropathology and may provide opportunities to develop novel therapeutic interventions for the treatment of PD [65]. Modulating CD4+T cell responses, including their metabolism and interactions with the brain, is a promising area of research for developing therapies for Parkinson’s disease. In-vitro strategies, such as adding specific amino acids (like L-arginine) or using metabolic modulators, help T cells develop into long-lived memory T cells with enhanced OXPHOS and fatty acid oxidation FAO are being explored [60]. Again, research works targeting metabolic checkpoints using inhibitors for enzymes like ACAT1 (acetyl-CoA acetyltransferase 1) can be implicated in the regulation of Aβ peptide, involved in AD. Blocking ACAT1 activity, beneficial effects are obtained, so it has been suggested that ACAT1 can be a potential new therapeutic target are being explored [3]. Furthermore, researchers are exploring the use of short-chain fatty acids like butyrate to enhance OXPHOS and function of Treg cells in order to make them more effective at suppressing inflammation [97].

Specific genes and factors being investigated as therapeutic targets for modulating T cell metabolism in neurodegeneration include those involved in the APOE pathway, T cell inhibitory receptors like PD-1, and key metabolic signaling molecules such as mTOR, HIF-1α and AMPK. For instance, work by Gedaly et al. [30] assessed the efficacy of the mTORC1/C2 inhibitor, AZD8055, in the manufacturing of clinically competent Treg cells and compared the effects with those induced by rapamycin (RAPA). The authors concluded that a distinct pattern of mTOR inhibition by AZD, compared with RAPA, induced mitochondrial stress response and dysfunction, impaired autophagy, and disrupted cellular bioenergetics, resulting in the loss of proliferative potential and suppressive function of Treg cells [30].

### Comparative metabolic profiles of CD4⁺ T cells in neurological diseases

Key metabolic traits from Parkinson’s disease (PD), multiple sclerosis (MS), and Alzheimer’s disease (AD) were compiled to clarify the function of CD4 T cell immunometabolism in various neurodegenerative situations and recorded [87]. The metabolic adaptability and dysfunction shared by CD4 T cells lead to inflammatory rather than regulatory consequences, even in the face of disease-specific neuropathology [96]. This comparability among diseases is shown in the table below (Table 1).

**Table 1:** Comparative immunometabolic profiles of CD4+T cells in PD, MS, and AD

Feature	Parkinson’s disease (PD)	Multiple sclerosis (MS)	Alzheimer’s disease (AD)
Effector T cell subsets	Th1, Th17	Th1, Th17	Th1, Th17
Tissue Infiltration	Substantia nigra, basal ganglia	Spinal cord, brainstem, periventricular lesions	Perivascular cortex, hippocampus
Cytokine Signature	IFN-γ, IL-17	IFN-γ, IL-17, GM-CSF	IFN-γ, TNF-α, IL-6
Effector Metabolism	↑ Glycolysis (mTORC1), ↑ Glutaminolysis	↑ Glycolysis, ↑ Glutaminolysis (robust mTORC1 activation)	↑ Glycolysis; disrupted mitochondrial energy production
Treg Metabolic Status	↓ FAO and OXPHOS; oxidative stress-induced instability	Mitochondrial dysfunction, impaired FAO, and low Foxp3 expression	Partially preserved FAO/OXPHOS; vulnerable in advanced stages
CNS Metabolic Stressors	Mitochondrial dysfunction, ↑ ROS	Hypoxia, glucose restriction, ↑ lactate, and inflammation	Amyloid burden, nutrient scarcity, ↑ ROS
Key Metabolic Pathways Affected	mTORC1 activation, ROS signaling, impaired Foxp3 regulation	mTORC1–AMPK imbalance, HIF-1α signaling	AMPK suppression, SIRT1 dysregulation, ROS overload
Functional Consequences	Enhanced neuroinflammation, dopaminergic neuron loss	Demyelination, BBB disruption, relapse progression	Microglial activation, synaptic dysfunction, and cognitive decline
Therapeutic Direction	Enhance Treg bioenergetics; block effector glycolysis	Glycolytic inhibition; FAO/OXPHOS reconstitution in Tregs	Promote Treg mitochondrial fitness; modulate glycolysis and ROS

A schematic overview of effector (Th1/Th17) and regulatory (Treg) CD4⁺ T cell phenotypes, their predominant metabolic pathways (glycolysis, FAO, OXPHOS), and their functional implications in the central nervous system spanning Parkinson’s disease, multiple sclerosis, and Alzheimer’s disease is shown in the table above. Arrows show pathways that are activated or suppressed. Important signaling nodes (mTOR, AMPK, Foxp3) and prominent stressors (e.g., ROS, hypoxia, glucose deprivation) are included in each illness setting to illustrate metabolic bottlenecks and potential treatment targets.

### Future perspective

The relationship between T cell metabolism and neurodegenerative diseases is becoming an increasingly important area of research [19]. Distinct subsets of CD4+T cells, such as Th1, Th2, Th9, and Th17, primarily rely on glycolysis for their energy production, whereas Treg cells are more dependent on lipid metabolism, particularly FAO [37]. This difference in metabolic pathways not only influences T cell function but also their fate in the context of neurodegeneration [89]. Recent studies have highlighted the dynamic metabolic adaptations that CD4+T cells undergo during the pathogenesis of neurodegenerative diseases like AD, PD, and MS [17]. These findings have opened up new possibilities for targeting T cell metabolism as a potential therapeutic strategy in these conditions. Since the immune response, particularly the activation and differentiation of T cells, plays a significant role in the progression of neurodegeneration [17], modulating these metabolic pathways may help in shifting the immune environment from a proinflammatory state (mediated by Th1 and Th17 cells) toward an anti-inflammatory state (promoted by Tregs). For example, targeting the metabolic shift from glycolysis to FAO in T cells could inhibit the proinflammatory responses of Th1 and Th17 cells [53], which are known to contribute to neuronal damage in these diseases. Meanwhile, promoting the metabolic pathways that favor Treg differentiation and function could enhance the neuroprotective, anti-inflammatory response, helping to protect neurons from degeneration [55]. While these findings are promising, much remains to be explored, particularly in understanding the specific metabolic alterations in Th17 and Treg cells in the context of neurodegenerative diseases. The complex interplay between T cell metabolism, immune responses and neurodegenerative pathology presents both challenges and opportunities for therapeutic intervention.

In the future, research into CD4+T cell metabolism in neurodegenerative diseases is expected to reveal novel mechanisms that drive T cell fate and function. This may lead to the identification of new targets for therapeutic interventions aimed at reprogramming T cells to either reduce neuroinflammation or promote neuroprotection. By better understanding how metabolic reprogramming influences T cell behavior in neurodegeneration, it may be possible to design more effective treatments that could slow disease progression and improve outcomes for patients suffering from diseases like AD, PD, and MS. In summary, the emerging evidence of CD4+T cell metabolic adaptations in neurodegenerative diseases underscores the importance of metabolic pathways in shaping immune responses. Further research in this field holds the potential to uncover novel therapeutic strategies that could offer new hope for managing these devastating diseases.

## Conclusion

The relationship between metabolism and immunity is an emerging field, with immune cell functions being closely tied to their metabolic states. Different CD4+T cell subsets, such as Th1, Th2, Th17, and Treg cells, rely on different metabolic processes. Th1, Th2, and Th17 cells are more glycolytic, while Treg cells depend on lipid metabolism and OXPHOS. This metabolic diversity offers a potential opportunity for selective regulation of immune cells. By shifting activated T cells from glycolysis to FAO, Treg function can be enhanced. This metabolic reprogramming strategy is being explored for its potential in treating neurodegenerative diseases, with a focus on immunomodulation through specific metabolic regulation. Further research is needed to fully elucidate the interplay between CD4+T cells, immunometabolism, and neurodegeneration, which may lead to novel therapeutic strategies.

## Data Availability

Not applicable.

## Abbreviations

Aβ: Amyloid beta
AD: Alzheimer disease
AMPK: AMP-activated protein kinase
BBB: Blood brain barrier
CNS: Central nervous system
CP: Choroid plexus
EAE: Experimental allergic encephalomyelitis
FAO: Fatty acid oxidation
FOXP3: Forkhead box protein P3
HIF-1α: Hypoxia-inducible factor 1-alpha
MS: Multiple sclerosis
MVB: Multivesicular bodies
mTOR: Mammalian target of rapamycin
ND: Neurodegenerative disease
NDE: Neuron derived exosomes
PD: Parkinson’s disease
ROS: Reactive oxygen species
RNS: Reactive nitrogen species
TCA: Tricarboxylic cycle
Treg: T regulatory T cells
